# Supplementary figures and images for: Activity-based CRISPR scanning uncovers allostery in DNA methylation maintenance machinery
Source: eLife. 2023 Feb 10;12:e80640. doi: 10.7554/eLife.80640 (PMC9946446; doi:10.7554/eLife.80640)

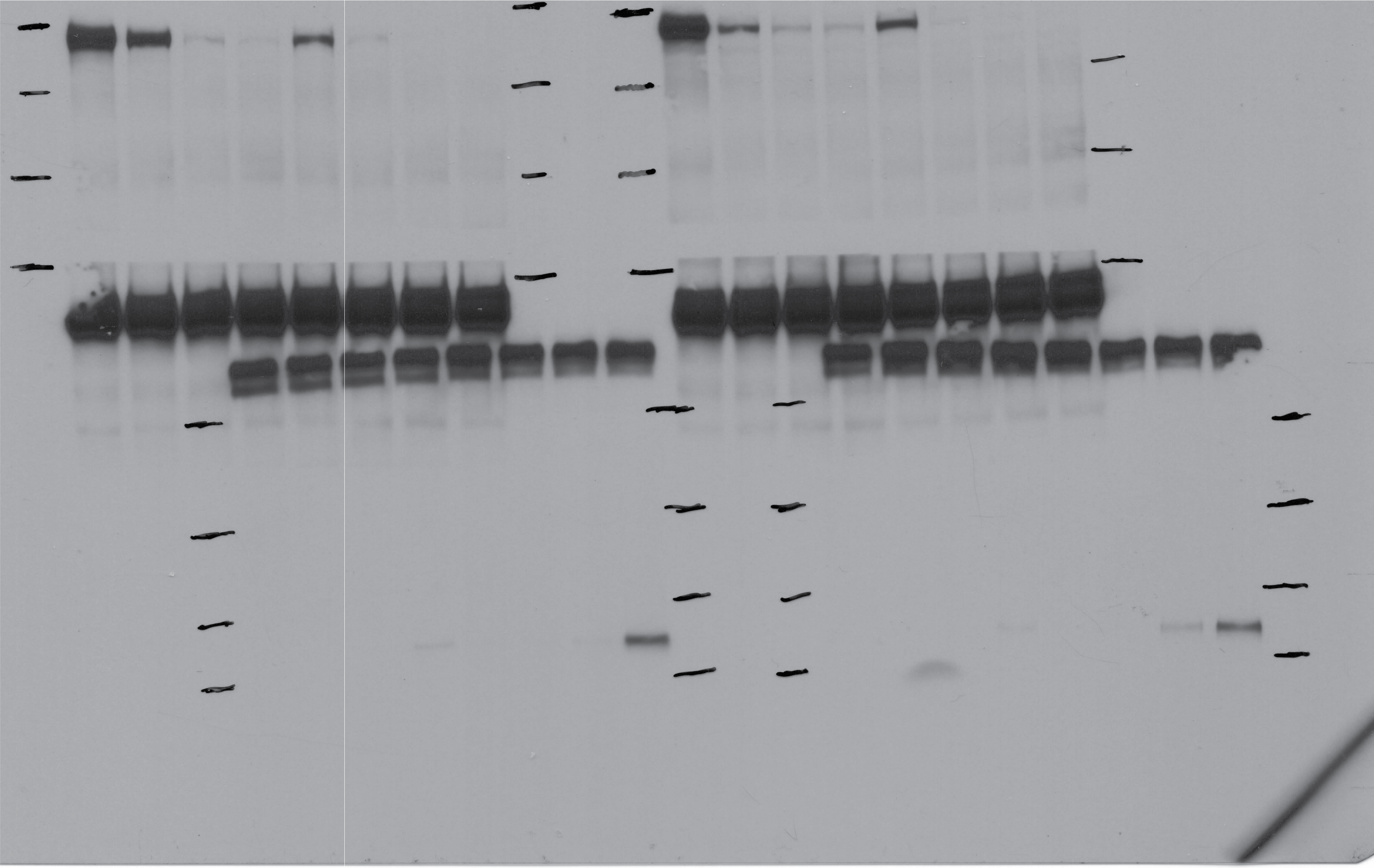

Supplement: Figure 1—figure supplement 1—source data 1. [file elife-80640-fig1-figsupp1-data1.zip › Figure 1-figure supplement 1-source data 1.png]

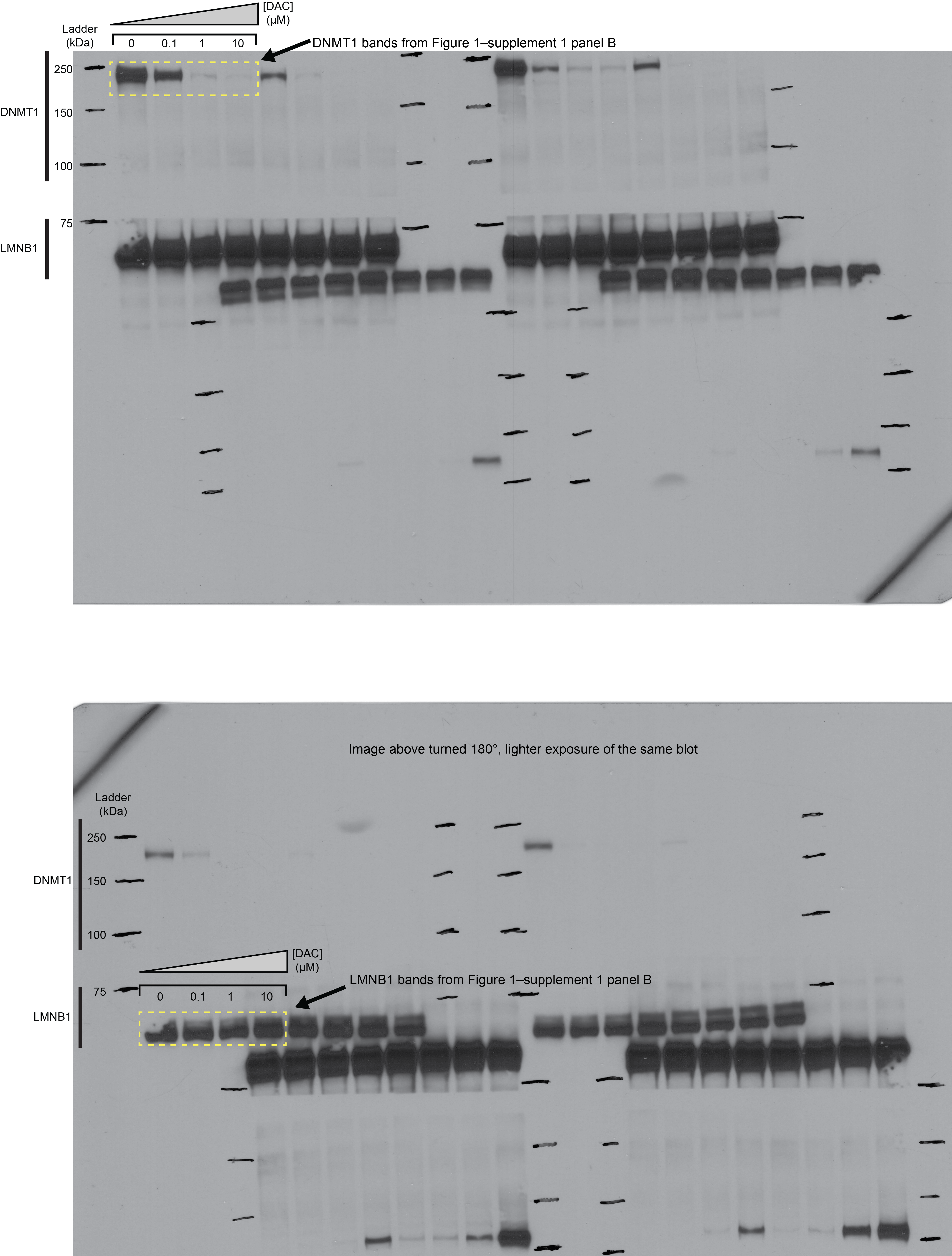

Supplement: Figure 1—figure supplement 1—source data 2. [file elife-80640-fig1-figsupp1-data2.zip › Figure 1-figure supplement 1-source data 2.png]

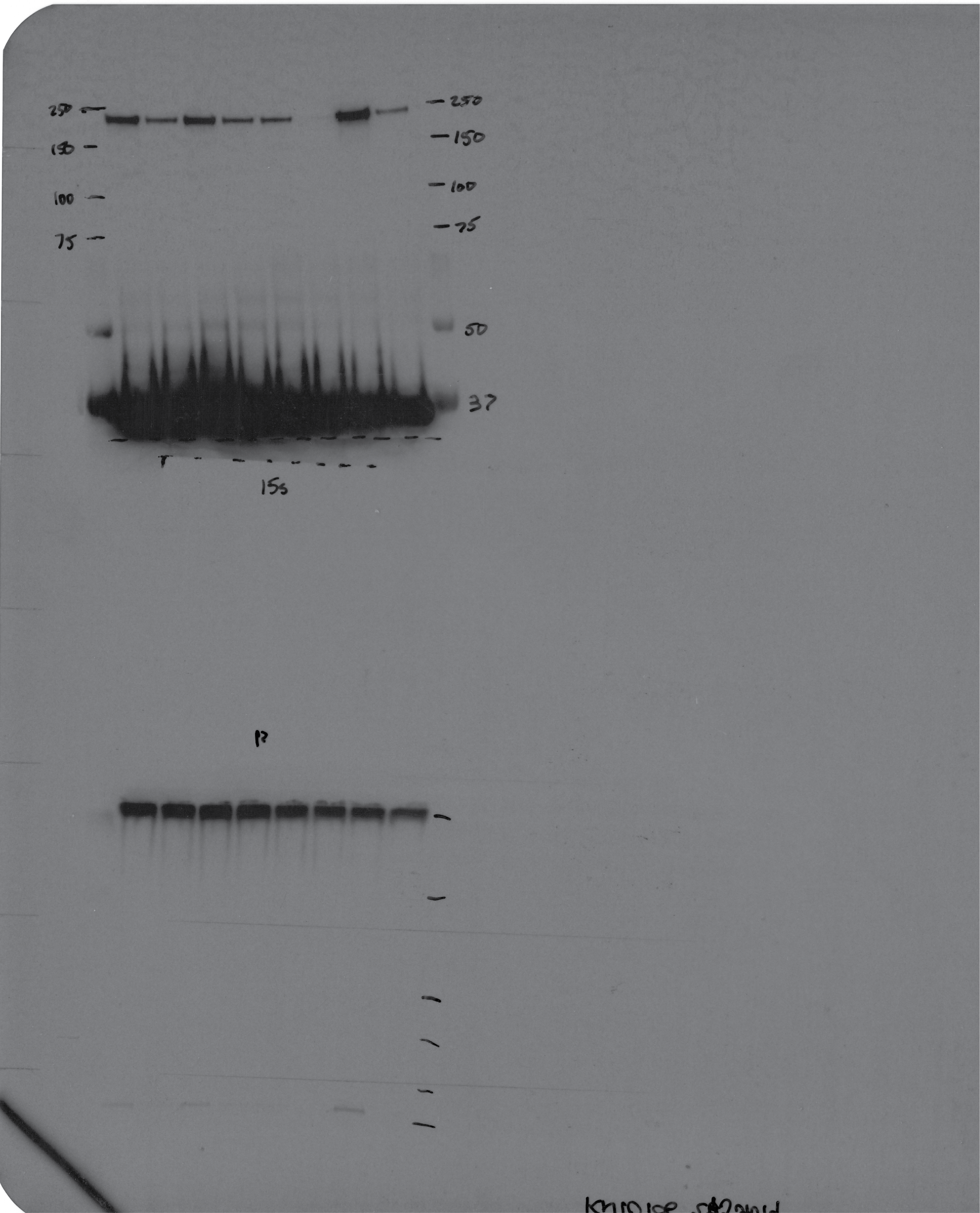

Supplement: Figure 3—figure supplement 1—source data 1. [file elife-80640-fig3-figsupp1-data1.zip › Figure 3-figure supplement 1-source data 1.png]

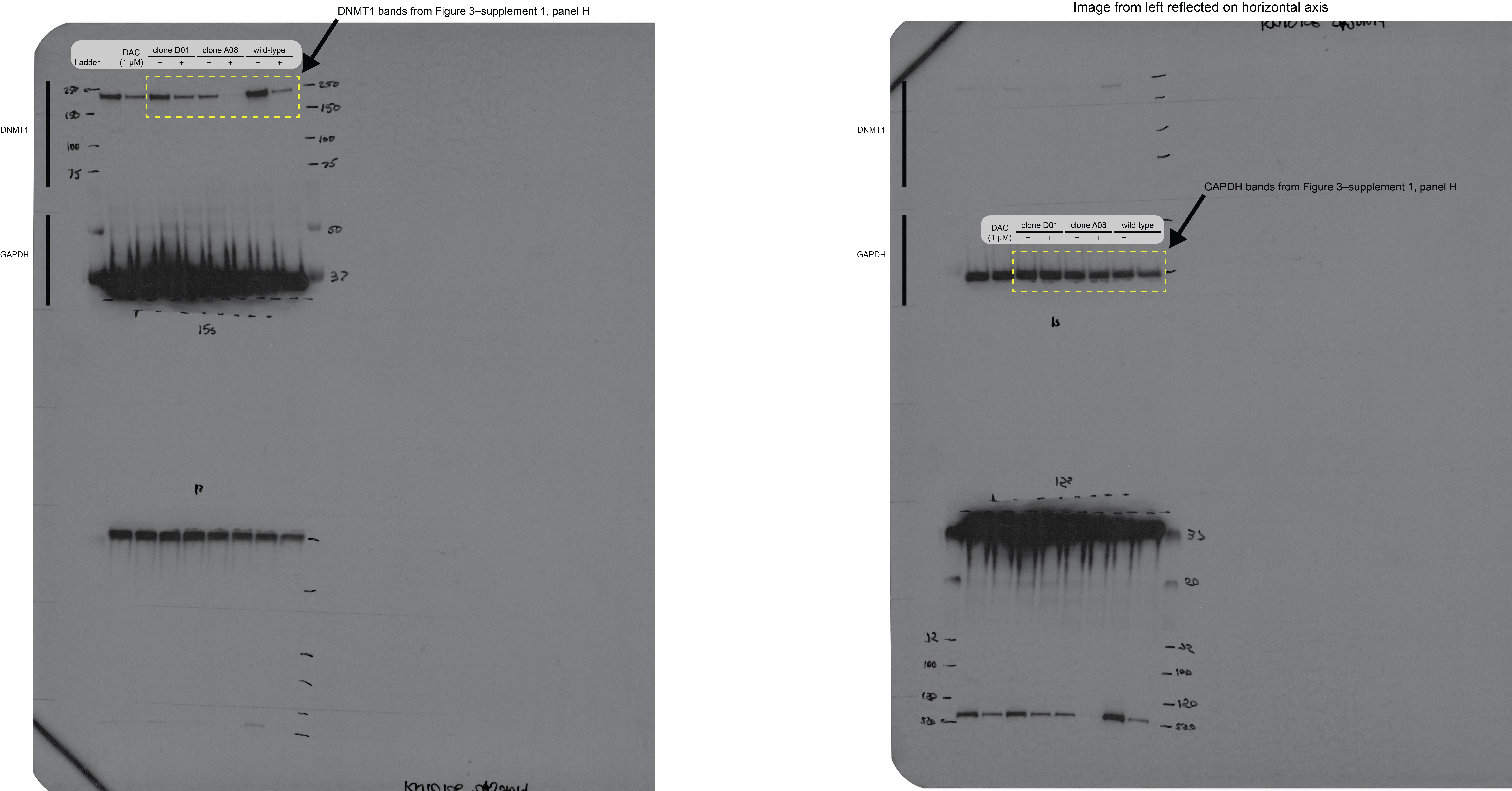

Supplement: Figure 3—figure supplement 1—source data 2. [file elife-80640-fig3-figsupp1-data2.zip › Figure 3-figure supplement 1-source data 2.png]

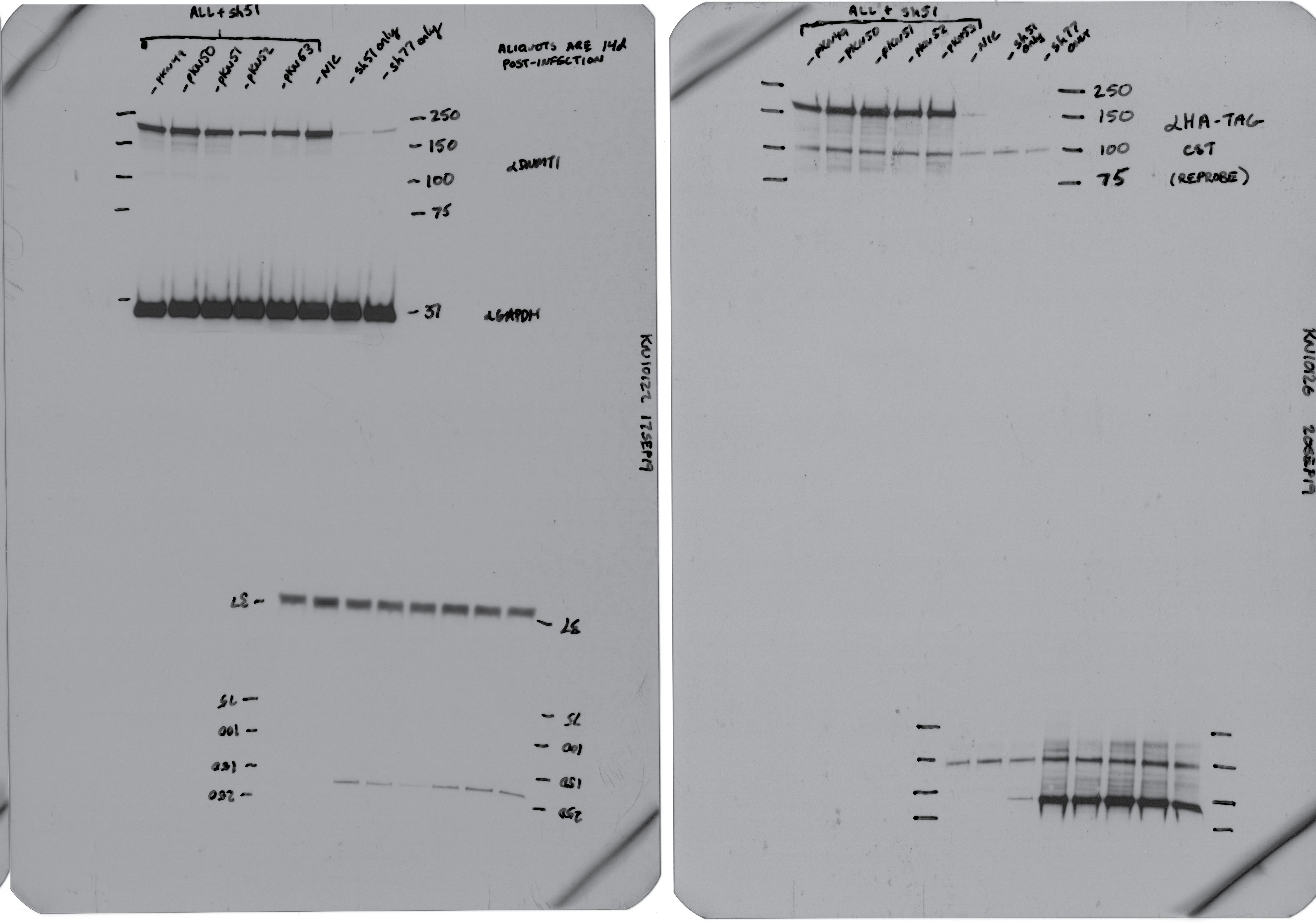

Supplement: Figure 3—figure supplement 1—source data 3. [file elife-80640-fig3-figsupp1-data3.zip › Figure 3-figure supplement 1-source data 3.png]

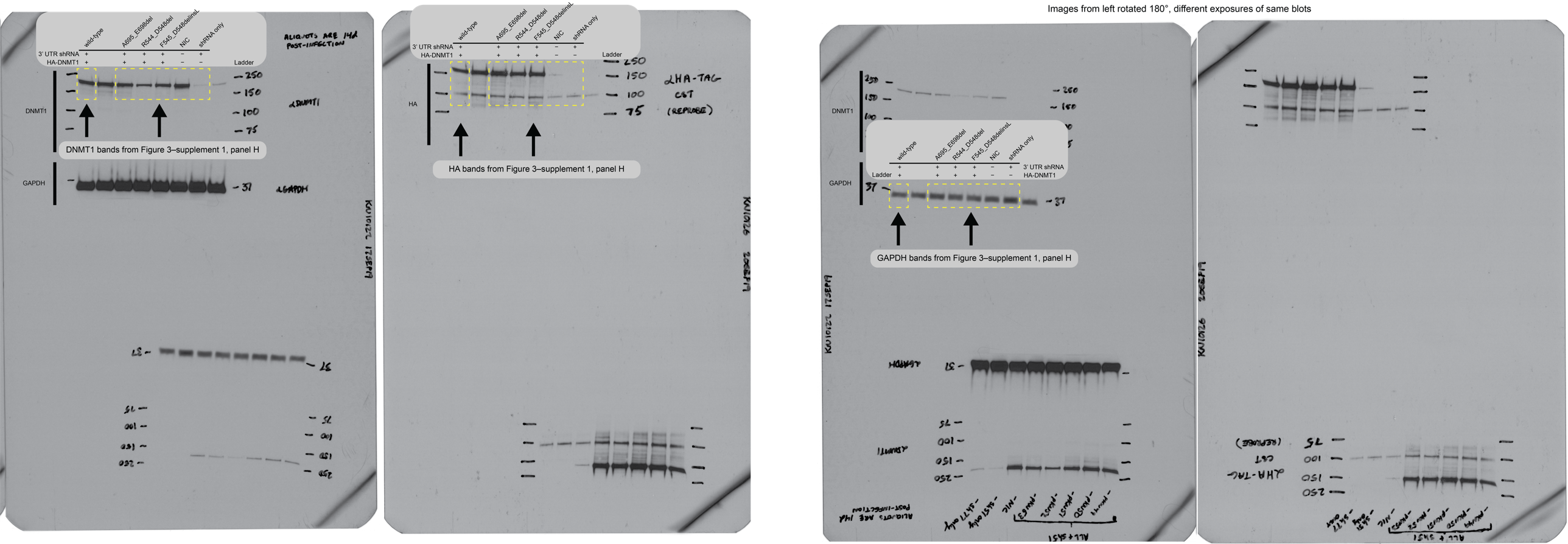

Supplement: Figure 3—figure supplement 1—source data 4. [file elife-80640-fig3-figsupp1-data4.zip › Figure 3-figure supplement 1-source data 4.png]
